# Exploring the Association Between Positive and Negative Social Support and Spiritual Well-Being: Results from the National Survey of American Life

**DOI:** 10.3390/ijerph22111660

**Published:** 2025-11-01

**Authors:** Shaila M. Strayhorn-Carter, Brook E. Harmon, Latrice C. Pichon, Michelle Y. Martin

**Affiliations:** 1 School of Health and Applied Human Sciences, University of North Carolina Wilmington, Veteran’s Hall 2506K, 601 S. College Rd., Wilmington, NC 28403, USA; 2 Nutrition and Foods Program, Department of Nutrition and Health Care Management, Appalachian State University, Leon Levine Hall of Health Sciences, Boone, NC 28608, USA; harmonbe1@appstate.edu; 3 Division of Social and Behavioral Sciences, School of Public Health, University of Memphis, 3825 Desoto Ave., Memphis, TN 38152, USA; lcpichon@memphis.edu; 4 Department of Preventive Medicine, College of Medicine at the University of Tennessee Health Science Center, 221 Doctors Office Building, Memphis, TN 38163, USA; mmart126@uthsc.edu

**Keywords:** quality of life, chronic illness, social network, African American, Caribbean Black

## Abstract

Previous studies have found that support that is uplifting in nature (i.e., positive social support) can have a positive influence on the spiritual well-being of individuals with chronic diseases. However, few studies have explored positive and negative social support’s (i.e., the individual receiving the support feeling unsupported) impact. The purpose of this study is to assess the relationship between positive and negative social support and spiritual well-being among individuals of African descent with chronic illnesses. Survey items that focused on positive and negative social support as well as spiritual well-being were obtained from a secondary dataset, the National Survey of American Life. Missing imputation models were adjusted by demographic characteristics (gender, age, income, education, marital status, employment, length of stay in the U.S., insurance, and religious service attendance). Findings from the analysis revealed a positive association between positive social support and spiritual well-being (β: 0.07, SE: 0.01, *p* < 0.0001). No significant associations were observed between negative social support and spiritual well-being (β: 0.01, SE: 0.01, *p* = 0.51). Future researchers should continue to explore the impact of social support on the spiritual well-being of individuals of African descent through the implementation of a culturally tailored program designed to reduce chronic diseases within this population.

## 1. Introduction

It is estimated that 6 out of every 10 Americans are living with a chronic illness [1]. Chronic illnesses are defined as an illness that often progresses slowly and requires management over time [2]. Diabetes, cancer, stroke, and hypertension are just a few examples of chronic illnesses that are commonly diagnosed among American adults [3]. Such illnesses are capable of dramatically altering an individual’s physical health, which in turn can reduce their quality of life (QOL) [4]. Studies have recently begun to consider the impact that chronic illnesses may have on one specific QOL domain, spiritual well-being [5]. Spiritual well-being focuses on an individual’s purpose or meaning in life [6]. In addition to this, it is also conceptualized as an individual’s relationship with a higher being such as God [7,8]. A previous systematic review has indicated that the spiritual well-being among individuals with chronic diseases can impact various health outcomes such as physical functioning, depression, and sadness [9]. However, the participants within the study populations of this review were not representative of racially marginalized communities. Furthermore, little if any research has been conducted that assesses the spiritual well-being of a racially diverse population with chronic illnesses who identify as being of African descent.

### Literature Review

Spiritual well-being is a particular quality of life domain that has been shown to greatly impact individuals of African descent. The inclusion of participants of African descent (i.e., African American and/or Caribbean Black) in particular is imperative as approximately 90% of individuals of African descent have reported they believe in the existence of God [10]. Moreover, having a strong spiritual foundation [11] is highly influential among individuals of African descent. For example, a strong spiritual well-being among individuals of African descent has been attributed to health behaviors (i.e., substance use [12]) and symptoms of mental health [13]. It is the spiritual well-being of individuals of African descent that has also been shown to withstand race-based trauma and systematic inequalities [14]. For example, spiritual well-being among individuals of African descent has been shown to serve as a coping mechanism in managing institutional racism [15]. However, there remains uncertainty regarding the specific factors that may impact the spiritual well-being of individuals of African descent. Social support is a factor that is believed to be one of the strongest predictors of spiritual well-being. However, research is currently limited regarding the exploration of the relationship between factors of social support and spiritual well-being among individuals of African descent.

Social support is a concept in which an individual perceives that social resources are readily available to them through formal and informal social networks [16]. Members of an individual’s social network, who provide support, can also play a role helping an individual feel a strong sense of belonging and enhance their meaning of life [17]. In addition to this, social support can also serve as a buffer against stress [18], which may provide individuals with chronic illnesses, including various cancers, with more opportunities to better focus on their lives. The concept of social support serving as a buffer against stress is a theory known as the Stress Buffering Hypothesis [18]. This hypothesis suggests that perceived positive support can improve specific components of an individual’s quality of life [18]. Alternatively, perceived negative support can enhance stress thus leading to worse health outcomes [18]. The authors of this study believe that the stress buffering hypothesis can provide additional insight into the impact of both positive social support and negative social support on the quality of life among individuals previously diagnosed with chronic illnesses.

Positive social support is defined as social interactions that allow an individual to feel affirmed [19]. Alternatively, negative social support is defined by the manner in which an individual feels unsupported despite the providers’ intentions [19]. As a result, positive social support is believed to assist in buffering stress, while negative social support can aid in amplifying stress [20]. While the relationship between positive support and spiritual well-being has been previously explored [21], the impact of negative social support on this specific quality of life domain has not been thoroughly assessed. Prioritizing the role of negative social support on the spiritual well-being of individuals with chronic diseases would allow for a better understanding of how potentially harmful social interactions may worsen their symptoms or negatively impact their life’s purpose. Research is also limited regarding the association between social support and spiritual well-being among individuals with various chronic illnesses such as diabetes, cardiovascular disease, cancer, or lung disease. Exploring the impact of social support on the spiritual well-being among individuals of African descent is essential as the members of this population often have multiple chronic illnesses to manage [22]. Furthermore, individuals of African descent with a history of a disease such as cancer have exhibited improved quality of life due to enhanced social support [23]. However, such research is limited in exploring the potential associations between both positive and negative social support and the spiritual well-being of individuals of African descent with various chronic diseases, including cancer. Therefore, the purpose of this study is to assess the relationship between both positive and negative social support and spiritual well-being among individuals of African descent with chronic illnesses. By expanding this literature to include a population of individuals with various chronic illnesses, future researchers can acquire a more comprehensive understanding of the influence of social support on the spiritual well-being of this population.

Furthermore, we recognize that the findings of this study are essential given the ongoing psychosocial stressors currently impacting individuals of African descent. Individuals of African descent are currently expressing stress due to racism, discrimination, and acculturation post the COVID-19 pandemic [24,25]. Such stress can have severe negative health consequences for individuals with pre-existing chronic health conditions. By understanding how both positive and negative social support influence spiritual well-being, this study can provide additional insight into key coping mechanisms within this high-risk population. Moreover, by encouraging improvements in social support, this study will be able to provide culturally grounded strategies that can strengthen spiritual well-being, which in turn can improve the quality of life for individuals within this population.

## 2. Materials and Methods

### 2.1. Participants

All participants from this study were derived from the secondary dataset, the National Survey of American Life: Coping with Stress in the 21st Century (NSAL) [26]. The objective of this study was to assess psychological disorders among non-Hispanic whites and individuals of African descent [26,27]. Recruitment strategies and data collection procedures can be found within previously reported manuscripts [26,28]. In brief, findings from the NSAL are based on 6082 face-to-face interviews among Americans who identified as being 18 years of age or older, were able to speak English, were residents of one of the 48 American states, and self-identified as being either non-Hispanic white, African American, or Caribbean Black [29]. Participants self-identified as being Caribbean black if they self-identified as Black, reported that a parent/grandparent was born in a Caribbean-area country, and/or identified as being of West Indian or Caribbean descent [26]. A total of 3570 were African American, and 1623 were Caribbean Black during the data collection period of February 2001 to June 2003 [26]. Individuals who identified as African American or Caribbean Black were included in the study’s sample. By including both non-Hispanic whites and individuals of African descent within this study sample, our research team hopes to showcase the racial differences in social support factors and spiritual well-being.

For this study, participants were also considered eligible if they responded “yes” to having one of the fifteen chronic illnesses: chronic lung disease, sickle cell disease, heart trouble, cancer, diabetes, liver problem, osteoporosis, high blood pressure, arthritis, ulcers, stroke, glaucoma, asthma, blood circulation problems, and/or kidney problems. This list was chosen after reviewing studies that defined their chronically ill study sample using these specific chronic illnesses [30,31]. All data collection was approved by the Institutional Review Board of the University of Michigan [32]. During the time of the secondary data analysis, the Institutional Review Board of the University of Memphis did not require any additional approvals.

### 2.2. Measurements

Independent Variables. Twelve items from the NSAL dataset were related to positive and negative social support. Six items measured positive and negative social support from family (three items for positive social support and three items for negative social support). Similarly, six items also measured positive and negative social support from church members. An example of one of the positive social support items is “How often do your family members make you feel loved and cared for?” Alternatively, an example of one of the negative social support items is “How often do the people in your church make too many demands on you?” Each of these items was derived from previously developed scales and had a Cronbach’s alpha ranging from 0.64–0.86 [33,34]. The categories for each item were as follows: very often, fairly often, and never/not too often. To encompass all items related to positive social support, the six items from the survey were summed to create one variable. These items focused on positive social support from family and church members. Likewise, all six items that measure negative social support scores were also summed. These items were converted into continuous items and displayed a range of 6–18. An example of one of the items utilized within the positive social support variable, “How often do the people in your church make you feel loved and cared for?” with the responses being “very often”, “fairly often”, “not too often enough”, and “never”. For the purpose of this study, categorical social support items were converted into continuous items by coding “very often” as 3, combining “fairly often” with “not too often enough” then coding it as 2, and coding “never” as 1. The range for both positive and negative social support was between 6–18. High scores indicated either more frequent positive or negative social support. The current dataset does not include additional items from existing psychometric surveys related to negative and positive social support.

Dependent Variables. After reviewing psychometrically sound instruments designed to assess an individual’s spiritual well-being [35,36], two items within the NSAL dataset were identified (“Importance of spirituality in your life?” and “How spiritual are you?”) as reflecting a participant’s spiritual well-being similar to previous psychometric instruments. These items were combined into one general category measuring spiritual well-being. The range for this item was 2–8 with a higher score indicating better spiritual well-being. The 2001–2003 NSAL dataset does not possess additional items regarding spiritual well-being that reflect existing psychometric survey tools.

Analytical Strategy. All descriptive statistics and multivariate analyses were conducted using SAS 9.4 [37]. Two complete case linear Ordinary Least Squares (OLS) regressions were also conducted for both positive and negative social support. The researchers of this study also chose to assess the pattern of missingness within this study by conducting a Little’s Missing Completely at Random (MCAR) test [38]. The statistical program used to assess the pattern of missingness was IBM SPSS Statistics 25 [39]. After conducting this test, the *p*-value was less than 0.05 (χ^2^= 155.05, *p* < 0.0001). Such findings confirm that the data are not missing completely at random and multiple imputation analyses should be conducted to analyze the missingness within the dataset. As a result, we focused our findings on the beta estimates, standard deviations, and *p*-values from the multiple imputation analyses. These analyses also included the previously mentioned demographic variables as confounders.

## 3. Results

All demographic characteristics are depicted in Table 1. Of the 2758 study participants, the majority were female (n = 1845, 66.9%), between the ages of 45 to 59 (n = 829, 30.1%), reported an annual household income of $0–$19,999 (n = 1209, 43.8%), had at least 12 years of education (n = 916, 33.2%), fell in the category of being divorced/widowed/separated (n = 1039, 37.7%), were employed (n = 1616, 58.6%), born in the U.S. (n = 2187, 79.3%), and insured (n = 2302, 83.5%). Many study participants also indicated that they attended a religious service at least a few times a month (n = 1010, 36.6%). Study participants reported high levels of positive social support from family and church members (Mean: 13.2, SD: 3.0). Moderate levels of negative social support from family and church members were also observed among study participants (Mean: 7.3, SD: 2.1). Participants also reported high spiritual well-being scores (Mean: 7.2, SD: 1.0).

All models controlled for the following categorical demographic variables: age, gender, years of education, annual household income, employment status, marital status, and insurance coverage. The frequency of religious service attendance was also controlled for given its impact on social support from church members [33]. The multiple imputation revealed a positive association between positive social support and spiritual well-being among study participants (β: 0.07, SE: 0.01, *p* < 0.0001) (see Table 2). No statistically significant association was observed between negative social support and spiritual well-being (β: 0.01, SE: 0.01, *p* = 0.51). No differences in the magnitude of association were observed between the linear regression and the multiple imputation analyses. However, a stronger magnitude of association was observed among positive social support compared to negative social support. More specifically, for every unit increase in positive social support, there is a 0.07 increase in spiritual well-being among all study participants.

## 4. Discussion

The focus of this study was to assess the associations between both positive and negative social support and spiritual well-being among individuals of African descent with chronic illnesses. The findings of this study revealed positive social support was positively associated with the spiritual well-being of individuals of African descent, who were previously diagnosed with various chronic diseases. However, no association was observed between negative social support and spiritual well-being among study participants.

The findings of this study suggest that as positive social support increases, the spiritual well-being of individuals of African descent with chronic illnesses also increases. Such findings are also similar to previous studies, which observed an association between positive social support and various quality of life domains among cancer survivors of African descent [40,41]. This association may be due to positive social support moderating the relationship between the stress of the study participants who were diagnosed with chronic diseases, including cancer, and their spiritual well-being. This concept is similar to the stress-buffering hypothesis proposed by Cohen and Wills, which suggested that positive social support from an informal source can buffer or moderate the relationship between stress (either psychological and environmental stress) and specific health outcomes [18].

The research team of this study also observed that approximately 44% of the study population identified as having a household income of less than $19,000 and 41.4% were unemployed during the time of data collection. Such personal-level factors may influence individuals to have hope despite their current financial circumstances. Such hope is believed to allow individuals experiencing various hardships to become more willing to receive support from members of their social network [42,43]. It is therefore a possibility that the concept of hope may also play a role in the relationship between positive social support and spiritual well-being within this study population as well.

Negative social support was not negatively associated with spiritual well-being. This lack of association may be due to ambiguity when defining negative social support. Negative social support has also been defined as having little if any support from members of an individual’s social network [44]. In addition to this, negative social support has also been operationalized by survey items that reflect feelings of loneliness among individuals with chronic illnesses [45]. While there is no universal definition for negative social support, it is encouraged that additional research be conducted on this concept, as it can provide additional insight into unsupportive social interaction that can be detrimental to an individual’s quality of life. By expanding research in negative social support, researchers can better inform culturally tailored public health interventions within high-risk populations.

Future researchers should consider creating a survey that assesses negative social support among individuals with chronic illnesses as a means of learning what items should and should not be used to operationalize this variable. The creation of such a tool is essential due to negative social support potentially producing more detrimental health outcomes compared to positive social support [46]. The development of further defining negative social support may also allow for improved understanding of its impact on various quality of life domains among cancer survivors of African descent. For example, negative social support has been associated with reduced mental well-being scores among African American breast cancer survivors [40]. Negative social support has also been associated with impacting specific mental well-being factors such as depression among Korean adults [47] and Chinese female cancer survivors [48]. Such findings encourage additional research that explores the relationship between negative social support and its potential negative impact on the quality of life domains, such as spiritual well-being.

### 4.1. Strengths and Limitations

There were several strengths and limitations of the study. First, this is the first study to date to assess the association between both positive and negative social support and spiritual well-being specifically among individuals of African descent with chronic illnesses. As a result, the findings of this research will allow for the implementation of culturally tailored programs that can promote effective social support strategies among African American diagnosed with various chronic diseases. We also recognize that the items used to measure the dependent and independent variables were not derived from psychometrically sound questionnaires and therefore, may not demonstrate reliability within future studies that utilize different study populations.

Due to this study being a cross-sectional study, both the causality and the direction of the association between the variables in question cannot be determined. The recruitment and data collection methodologies for the NSAL were conducted over twenty years ago. As a result, various demographic characteristics that may also influence the relationship between these variables (i.e., type of treatment/surgery received, year of diagnosis, etc.) were not collected during the data collection phase. We also recognize that the demographic characteristics of the study population may not reflect the existing population of individuals of African descent with pre-existing chronic conditions. While this dataset may be over twenty years old, it does not eliminate the importance of the influence of certain types of social support on the spiritual well-being of individuals of African descent. As a result, the findings of this study can provide a crucial point of comparison for future post-pandemic studies. Such comparisons between this study’s findings and post-pandemic findings can allow future researchers to determine if both consistency and reliability of the results can be achieved using similar target populations. Lastly, it is unknown whether the findings of this study may be generalizable to all individuals of African descent.

### 4.2. Future Implications

Incorporating positive social support within future health interventions may prove to be an effective strategy for improving both the spiritual well-being and overall QOL of individuals of African descent with chronic illnesses post-pandemic, such as cancer. However, a longitudinal study is recommended to understand the influence of positive social support on the spiritual well-being of individuals of African descent with chronic illnesses, including cancer, over time. The findings from this longitudinal study can play a key role in aiding future researchers and clinicians in implementing culturally tailored interventions that are designed to promote positive social support among cancer survivors of African descent, with the hope of improving such quality of life domains as spiritual well-being. The authors of this study would also recommend conducting mediation and moderation analyses to determine if additional psychosocial factors (i.e., types of stress) may have an indirect impact on the relationship between positive/negative social support and spiritual well-being among a racially diverse study sample of adults with various chronic illnesses.

## 5. Conclusions

The findings of this study revealed that positive social support plays an influential role in improving the spiritual well-being of individuals of African descent with chronic illnesses. Positive social support can aid in providing individuals with various chronic illnesses with a renewed sense of purpose and meaning of life. By promoting positive social support, future providers may be able to directly enhance the spiritual well-being of their patients, which may also aid in improving both their overall quality of life and their survivorship.

## Figures and Tables

**Table 1:** Demographic Characteristics of Study Population from the NSAL Findings.

	Total (n = 2758) N (%)
**Sex ^a^**	
Male	913 (33.1)
Female	1845 (66.9)
**Age ^a^**	
18–29 years	392 (14.2)
30–44 years	817 (29.6)
45–59 years	829 (30.1)
60 years or more	720 (26.1)
**Household Income ^a^**	
$0–$19,999	1209 (43.8)
$20,000–$39,999	764 (27.7)
$40,000–$59,999	378 (13.7)
≥$60,000	407 (14.8)
**Years of Education ^a^**	
0–11 years	813 (29.5)
12 years	916 (33.2)
13–15 years	597 (21.6)
≥16 years	432 (15.7)
**Marital Status ^a^**	
Married/cohabiting	1018 (36.9)
Divorced/widowed/separated	1039 (37.7)
Never married	701 (25.4)
**Employment Status ^a^**	
Employed	1616 (58.6)
Unemployed	1142 (41.4)
**Length of Stay in the U.S. ^a^**	
U.S. Born	2187 (79.3)
<5 years	33 (1.2)
6–10 years	56 (2.0)
11–20 years	134 (4.9)
≥21 years	312 (11.3)
Missing	36 (1.3)
**Insurance Coverage ^a^**	
Insured	2302 (83.5)
Not Insured	456 (16.5)
**Religious Service Attendance ^a^**	
Less than once a year	767 (27.8)
A few times a year	634 (23.0)
A few times a month (1 to 3 times)	1010 (36.6)
At least once a week (1 to 3 times)	173 (6.3)
Nearly every day (4 or more times a week)	174 (6.3)
**Positive Social Support ^b^**	13.2 ± 3.0
**Negative Social Support ^b^**	7.3 ± 2.1
**Spiritual Well-being ^b^**	7.2 ± 1.0

^a^ Values represent n (%). ^b^ Values represent the mean ± standard deviation. Higher scores represent either more positive social support, negative social support, or spiritual Well-being.

**Table 2:** Unstandardized Beta Coefficients for the Association between Positive and Negative Social Support and Spiritual Well-being (n = 2758) ^a^.

	Model 2 ^b^
	Beta (SE)
**Positive Social Support**	0.07 (0.01) ***
**Negative Social Support**	0.01 (0.01)

*** *p* < 0.0001. ^a^ Models are fully adjusted for: gender (reference: males), age (reference: less than 29 years), income (reference: less than $19,000), education (reference: between 0–11 years), marital status (reference: married/cohabiting), employment (reference-employed), length of stay in the U.S. (reference: U.S. born), insurance (reference: insured), and religious service attendance (less than once a year/a few times a year). ^b^ Coefficients from the model are derived from multiple imputation analyses.

## Data Availability

Restrictions apply to the availability of these data. Data were obtained from Inter-university Consortium for Political and Social Research and are available upon consulting one of the PIs of this project (i.e., James Jackson, Cleopatra Caldwell, David Williams, Harold Neighbors, Randolph Nesse, Robert Taylor, and Steven Trierweiler) at https://www.icpsr.umich.edu/web/ICPSR/studies/27121 (accessed on 31 August 2018) with the permission of Inter-university Consortium for Political and Social Research.
